# Association Between Sedentary Behavior and Body Image Distortion Among Korean Adolescents Considering Sedentary Purpose

**DOI:** 10.3390/children13010020

**Published:** 2025-12-22

**Authors:** Suin Park, Heesoo Lee, Wanhyung Lee, Mi-Jeong Lee

**Affiliations:** 1 Kangbuk Samsung Hospital, Seoul 03181, Republic of Korea; psuin427@naver.com; 2 Graduate School of Public Health, Gachon University, Incheon 21565, Republic of Korea; 3 Department of Preventive Medicine, College of Medicine, Chung-Ang University, Seoul 06974, Republic of Korea; 4 Department of Nursing, Andong Science College, Andong 36616, Republic of Korea

**Keywords:** adolescents, sedentary behavior, body image, distortion, physical inactivity

## Abstract

**Highlights:**

**What are the main findings?**

- Prolonged sedentary behavior was associated with higher odds of body image distortion in adolescents, with this pattern driven mainly by girls.
- Educational sedentary time (study- and school-related sitting) showed a stronger link to body image distortion than non-educational sedentary time.

**What are the implications of the main findings?**

- Reducing prolonged sitting for educational purposes and integrating active study routines may help prevent body image distortion, especially among female students.
- School- and community-based programs should address both sedentary time and body image in adolescent health promotion, with particular attention to academic pressure in girls.

**Abstract:**

Background: Sedentary behavior in adolescents is a major pediatric health concern. Prolonged inactivity can negatively affect physical and mental health, potentially leading to body image distortion, especially among adolescents. This study aims to explore the relationship between sedentary behavior and body image distortion in adolescents, considering sedentary purpose and sex differences. Methods: This study analyzed data from the 2021–2023 Korea Youth Risk Behavior Survey (KYRBS), comprising 150,025 middle and high school students. Sedentary time was self-reported as the average daily sedentary duration over 7 days. Body image distortion was defined as a discrepancy between the body mass index and perceived body image of participants. Data analysis included descriptive statistics, chi-square tests, and logistic regression, stratified by sex and sedentary purpose. Increased sedentary behavior was significantly associated with body image distortion. Results: Among female adolescents, educational sedentary time had a stronger effect on body image distortion (odds ratio: 1.06, 95% confidence interval: 1.02–1.09). In contrast, male adolescents showed no significant association. Conclusions: This study highlights the significant association between sedentary behavior and body image distortion in adolescents. Future research should further explore the long-term effects of sedentary behavior on the physical and mental health of adolescents.

## 1. Introduction

In modern society, physical inactivity and sedentary lifestyles have become serious health concerns in adolescents, largely because of the increased use of digital devices and academic pressure [1]. This phenomenon not only alters daily routines but also contributes to other issues such as obesity, metabolic syndrome, cardiovascular problems, and mental health challenges [2,3]. Adolescence is an important phase for both physical development and emotional growth, including building self-esteem [4]. Therefore, sedentary behavior warrants close attention for its potential negative effect on the physical and mental health of adolescents.

Body image distortion occurs when individuals perceive their bodies differently from objective reality [5,6]. Although the direct pathway from sedentary behavior to body image distortion is not fully established, several mechanisms have been proposed. First, prolonged sedentary time displaces physical activity, which is crucial for developing “functional body image”—an appreciation of what the body can do rather than how it looks [4]. Without this physical engagement, adolescents may focus disproportionately on esthetic appearance [7]. Second, sedentary behavior, especially involving screen time, exposes adolescents to idealized body images on social media, leading to upward social comparison and internalization of unrealistic thin ideals [8].

This effect is hypothesized to be stronger in female adolescents. Adolescent girls generally experience stronger sociocultural pressures toward thinness and are more sensitive to appearance-related evaluation and social comparison than boys [9]. In addition, academic stress and internalizing symptoms may be more strongly linked to body dissatisfaction among girls [10]. Therefore, the relationship between sedentary time especially educational sedentary time and body image distortion may be more pronounced among female adolescents.

In particular, South Korean adolescents face a unique environment characterized by intense academic competition. This often forces students to spend excessive amounts of time in sedentary behaviors for educational purposes, such as attending schools and private academies, while opportunities for physical activity are drastically reduced. Recent trends indicate that while the socioeconomic status of Korean families has improved, the physical activity levels of adolescents have stagnated or declined, whereas sedentary time continues to rise [11]. This specific cultural context where sedentary behavior is often mandated by educational demands rather than leisure choice requires distinct investigation.

Prior studies have reported associations between sedentary behavior and body image outcomes, yet most have treated sedentary time as a single construct without distinguishing sedentary purpose [3]. Moreover, evidence on whether these relationships differ by sex remains limited. Using nationally representative data from Korean adolescents, this study examined (1) the association between total sedentary time and body image distortion, (2) whether sedentary time for educational versus non-educational purposes shows differential associations, and (3) whether these associations vary by sex.

## 2. Materials and Methods

### 2.1. Data Collection and Study Participants

**Figure 1:**
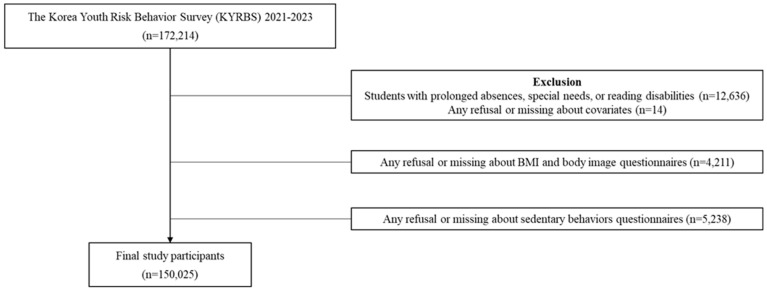
Flowchart of the selection process of participants.

This study analyzed primary data derived from the 17th to 19th Korea Youth Risk Behavior Survey (KYRBS) conducted between 2021 and 2023. This survey, conducted annually since 2005 by the Korea Disease Control and Prevention Agency (KDCA) under Article 19 of the National Health Promotion Act [12], collects anonymous self-reported data from middle and high school students covering various health behaviors, including dietary habits and physical activity. Out of 172,214 individuals included in the 2021–2023 KYRBS, some were excluded for the following reasons: 12,636 were absent or unable to self-report, 4211 did not respond to questions on body mass index (BMI) and body image perception, 5238 did not respond to questions on sedentary lifestyle, and 14 were either missing or refused to reply. Overall, 150,025 individuals (male: 76,776, female: 73,249) were included in the final analysis (Figure 1).

### 2.2. Sedentary Time

Participants reported the average daily duration of sedentary behavior over the past 7 days. Given the lack of a universally established clinical cutoff for excessive sedentary time in adolescents [13], and to examine potential dose–response relationships, participants were categorized into quartiles based on the distribution of the study population: 1–240 min (Q1, Reference), 241–300 min (Q2), 301–360 min (Q3), and ≥361 min (Q4). This data-driven approach minimizes the influence of outliers and allows for the identification of relative risk thresholds within the cohort.

For the sensitivity analysis, sedentary time was further stratified by purpose: educational (e.g., studying, class attendance) and non-educational (e.g., watching TV, gaming, resting). Quartiles for these sub-categories were independently calculated to reflect their distinct distributions.

### 2.3. Body Image Distortion

Body image distortion was defined as a discrepancy between the participant’s objective BMI status and their subjective body image perception, a method validated for use in large-scale adolescent surveys [14].

BMI was calculated from self-reported weight (kg) and height (m) and categorized according to the 2017 Korean National Growth Charts for children and adolescents: underweight (<5th percentile), normal (5th–84th percentile), overweight (85th–94th percentile), and obese (≥95th percentile). Subjective body image perception was assessed by the question, “How do you perceive your body type?” with five response options ranging from “Very thin” to “Very fat.”

Consistent with previous KYRBS studies, distortion was identified when a mismatch occurred (e.g., an underweight student perceiving themselves as “fat” or a normal-weight student perceiving themselves as “obese”) [14,15]. This categorical approach allows for the efficient identification of perceptual inaccuracies in a large population sample, although it does not capture the nuances of body composition (e.g., muscle mass).

### 2.4. Covariates

Covariates were selected a priori based on a conceptual framework derived from previous literature on adolescent health behaviors and body image, reflecting factors associated with both sedentary behavior and body image distortion [5,16,17,18].

These were categorized into three domains:

1. Sociodemographic factors: Sex, school level (middle/high school), academic achievement (high/middle/low), household economic status (high/middle/low), and living arrangement (with family/not).
2. Health-related behaviors: Regular breakfast consumption, frequent fast food consumption (≥3 times/week), alcohol consumption, and smoking status, which are known to cluster with sedentary lifestyles.
3. Psychological and Physical status: Perceived stress (high/low) and BMI category.

While we adjusted for perceived stress, other specific psychological scales (e.g., clinical depression diagnosis or media exposure) were not included in the primary adjustment model due to data availability in the KYRBS public dataset.

### 2.5. Statistical Analysis

All analyses were conducted using SAS version 9.4 (SAS Institute Inc., Cary, NC, USA). Demographics and health-related factors were analyzed using descriptive statistics to better understand the population. The relationship between sedentary lifestyle and behavior was assessed using chi-square tests, and a two-tailed *p*-value of <0.05 was considered statistically significant. For the multivariable analysis, logistic regression models were employed to estimate odds ratios (ORs) and 95% confidence intervals (CIs). Sedentary time was entered as a categorical variable (quartiles), to focus on the effect of high sedentary behavior, the lower three quartiles (Q1–Q3) were combined as the reference group, and the highest quartile (Q4) was analyzed as the exposure group. Models were adjusted for sex, school grade, academic achievement, household income, living with parents, regular breakfast consumption, fast food consumption, alcohol consumption, smoking, perceived stress, and BMI, stratified by sex and sedentary purpose.

## 3. Results

Figure 1 shows the participant selection process. Of the 172,214 individuals originally included in the 2021–2023 KYRBS data, 22,189 subjects were excluded due to refusal or missing data.

Given the large sample size, small differences across sedentary time groups frequently reached statistical significance. Therefore, emphasis was placed on the magnitude and direction of effect sizes, rather than *p*-values alone.

Table 1 summarizes participant characteristics according to quartiles of average daily sedentary time. Among the total 150,025 participants (76,776 males; 73,249 females), females had a significantly higher proportion in the highest sedentary quartile (≥361 min/day, 37.9%) compared to males (30.7%, *p* < 0.0001). Sedentary behavior increased significantly from middle school (28.5% in highest quartile) to high school students (41.0% in highest quartile, *p* < 0.0001). Additionally, significant variations in sedentary time were observed according to academic achievement, with higher-performing students showing greater sedentary time. Household income, living arrangements (living with parents or not), regular breakfast consumption, smoking, perceived stress, and BMI categories also significantly differed across sedentary behavior quartiles (all *p* < 0.0001).

Table 2 presents characteristics according to sedentary time specifically for educational purposes. Females demonstrated significantly greater educational sedentary behavior, with 38.6% in the highest quartile (≥481 min/day) compared to 27.8% of males (*p* < 0.0001). High school students were notably more sedentary for educational purposes (43.0% in highest quartile) than middle school students (24.7%, *p* < 0.0001). Additionally, high academic achievers (41.5%) and those from higher-income households (37.2%) spent significantly more sedentary time on educational activities. Similar to general sedentary behavior, significant differences were found according to living with parents, regular breakfast consumption, fast food consumption, alcohol intake, smoking, perceived stress, BMI, and body image distortion (all *p* < 0.0001).

Table 3 describes characteristics by quartiles of non-educational sedentary time. In contrast to educational sedentary time, males and females exhibited similar distributions in non-educational sedentary behavior, with 27.8% of males and 25.3% of females in the highest quartile (≥301 min/day, *p* < 0.0001). Middle school students spent more sedentary time on non-educational activities compared to high school students. Lower academic achievers and participants from lower-income households showed significantly greater non-educational sedentary behavior. Similar significant associations were observed for living with parents, breakfast consumption, fast food consumption, alcohol intake, smoking, perceived stress, BMI, and body image distortion, although with less pronounced differences compared to educational sedentary behavior (all *p* < 0.05).

Table 4 presents logistic regression results examining the association between sedentary behavior and body image distortion, stratified by sex and purpose of sedentary time. After adjusting for sex, school grade, academic achievement, household income, living arrangements, dietary habits, smoking, alcohol intake, perceived stress, and BMI, overall sedentary behavior ≥301 min/day was significantly associated with increased odds of body image distortion. Although the OR were statistically significant due to the large sample size, the magnitude of the association was modest. For female adolescents, educational sedentary time showed a marginal positive association with body image distortion (OR: 1.06, 95% CI: 1.02–1.09). In contrast, no significant association was observed in males (OR: 0.99, 95% CI: 0.96–1.03). This suggests a sex-specific pattern where sedentary behavior is more relevant to body image perception in females.

## 4. Discussion

This study examined the association between sedentary behavior and body image distortion in adolescents using nationally representative data. The results showed that prolonged sedentary behavior, particularly for educational purposes, was significantly associated with negative body image distortions, especially among female adolescents.

This finding aligns with previous studies highlighting the negative effects of prolonged sedentary behavior on both physical and mental health. For instance, one study linked sedentary behaviors in adolescents to higher anxiety and depression levels, which contribute to body image issues [19]. Similarly, sedentary lifestyles can increase body dissatisfaction and stress, which are key factors in body image distortion [8].

We observed that increased educational sedentary time was associated with slightly higher odds of body image distortion in girls (OR = 1.06, 95% CI: 1.02–1.09). While this effect size is small at the individual level, it may hold considerable public health relevance at the population level. As articulated by Geoffrey Rose in his seminal work on prevention strategies, “a large number of people at a small risk may give rise to more cases of disease than a small number of people at high risk” [20]. Applied to our context: although each adolescent exposed to high educational sedentary time has only a marginal increase in the odds of body image distortion, the ubiquity of sedentary behavior among Korean students means that this small effect, multiplied across the entire population, could contribute substantially to the overall burden of body image issues in adolescence. However, these small ORs must also be interpreted with caution. The attenuated effect sizes may partly reflect measurement limitations inherent in self-reported surveys, which tend to introduce non-differential misclassification and bias estimates toward the null. Moreover, reverse causation cannot be ruled out in this cross-sectional design.

Our study expands on these findings by differentiating between sedentary time for educational and non-educational purposes. While previous research often generalized sedentary behavior, our analysis revealed a stronger link between educational sedentary time and body image distortion [21]. This association was particularly pronounced among female adolescents, who face greater social pressure to excel academically and maintain a certain appearance. These findings align with research emphasizing the role of academic stress in shaping body image perceptions, as high academic demands can exacerbate body dissatisfaction [22]. However, no significant association was found between sedentary behavior and body image distortion in male adolescents, likely attributed to sex-specific social expectations and cultural factors influencing how adolescents perceive and react to their bodies.

Sedentary behavior among adolescents can lead to body image distortion through several mechanisms. It limits opportunities to engage in physical activity, which is key to developing a healthy body image [23]. Physical activity helps maintain weight and boosts mood, energy, and self-esteem. Without these benefits, sedentary individuals may feel inadequate or frustrated with their bodies, especially when comparing themselves to societal beauty standards [24]. Moreover, sedentary behavior often leads to weight gain, worsening negative body image perceptions [25]. Females are more vulnerable to the negative effects of sedentary behavior on body image because of societal expectations and cultural pressures to conform to ideals such as thinness [26]. Females often experience greater media influence and peer pressure regarding physical appearance, leading to heightened body image concerns [27]. Sedentary behavior related to educational purposes, such as studying, attending classes, or engaging in online lectures, uniquely contributes to body image distortion. The more sedentary time adolescents devote to educational activities, the less time they have for physical exercise or social activities that promote physical health and a positive body image [28]. This form of sedentary behavior, driven by academic pressure and the drive to succeed, can lead to stress and anxiety, which are closely linked to body dissatisfaction [29]. Adolescents may prioritize academic success over appearance, creating a disconnect between mental and physical health [30]. This imbalance can distort body image by reinforcing the notion that academic success and physical appearance are mutually exclusive [31].

While providing insights into the relationship between sedentary behavior and body image distortion among adolescents, this study has several limitations. Its cross-sectional design limits the ability to establish a causal relationship between sedentary behavior and body image distortion. Longitudinal studies are needed to examine how changes in sedentary behavior over time may influence body image perceptions and mental health outcomes. Additionally, the use of a large-scale, self-reported survey may introduce systematic measurement bias, including recall bias in sedentary time and reporting bias in height, weight, and body image perception. Such non-differential misclassification may have attenuated the observed associations. Although sedentary time was categorized using a data-driven quartile approach, these categories may not fully capture qualitatively meaningful differences in sedentary behavior, particularly with respect to posture, context, and intensity. Objective measurements of sedentary behavior using devices such as accelerometers or activity trackers could provide more accurate insights. Although the study differentiates between educational and non-educational sedentary time, it does not consider specific contexts, such as the type of academic tasks or non-educational activities (e.g., social media use, gaming, etc.). Understanding these specific contexts would provide insights into how different sedentary behaviors contribute to body image distortion. While the KYRBS employs a complex sampling design, potential clustering at the school level was not explicitly modeled in the present analyses. Although the large sample size and adjustment for multiple sociodemographic covariates may partially mitigate this issue, residual intraclass correlation cannot be excluded. BMI-based classification may imperfectly reflect adiposity during adolescence due to rapid growth and pubertal changes, potentially leading to misclassification of body image distortion. While several covariates were controlled for in the analysis, other potential confounders, such as depressive symptoms, anxiety, and detailed measures of media exposure, were not available or not included in the models. These factors may influence body image perception and could partially explain the observed associations. Future research using longitudinal designs, objective measures of sedentary behavior, and more nuanced assessments of psychological and social factors will be important for clarifying causal pathways. From a policy perspective, our findings suggest that interventions targeting sedentary behavior should consider not only duration but also context, while acknowledging that observed associations are modest at the individual level.

## 5. Conclusions

This study identified a link between sedentary behavior and body image distortion in adolescents. Those engaged in educational activities and female adolescents had a greater estimate of body image distortion. As sedentary behavior negatively associated both physical and mental health, including body image, it warrants further investigation. Future research should explore the long-term effects of sedentary behavior on body image and include clinical assessments to better understand these associations.

## Figures and Tables

**Table 1:** Participant characteristics related to weekly average daily sedentary behavior.

Characteristic	Total	Weekly Average Daily Sedentary Time								*p*-Value
		1–240 min/d		241–300 min/d		301–360 min/d		≥361 min/d		
Sex										<0.0001
Male	76,776	24,424	(31.8)	14,197	(18.5)	14,575	(19.0)	23,580	(30.7)	
Female	73,249	16,204	(22.1)	13,627	(18.6)	15,657	(21.4)	27,761	(37.9)	
School grade										<0.0001
Middle school	81,692	25,772	(31.6)	16,520	(20.2)	16,073	(19.7)	23,327	(28.5)	
High school	68,333	14,856	(21.7)	11,304	(16.5)	14,159	(20.8)	28,014	(41.0)	
Academic achievement										<0.0001
High	58,015	13,051	(22.5)	10,675	(18.4)	12,511	(21.6)	21,778	(37.5)	
Middle	45,370	12,406	(27.3)	8442	(18.6)	9123	(20.1)	15,399	(34.0)	
Low	46,640	15,171	(32.5)	8707	(18.7)	8598	(18.4)	14,164	(30.8)	
Household income										<0.0001
High	62,362	16,405	(26.3)	11,337	(18.2)	12,301	(19.7)	22,319	(35.8)	
Middle	70,857	19,281	(27.2)	13,319	(18.8)	14,638	(20.7)	23,619	(33.3)	
Low	16,806	4942	(29.4)	3168	(18.5)	3293	(19.6)	5403	(32.5)	
Living with parents										<0.0001
Yes	143,279	38,917	(27.2)	26,794	(18.7)	28,989	(20.2)	48,579	(33.9)	
No	6746	1711	(25.4)	1030	(15.3)	1243	(18.4)	2762	(40.9)	
Regular breakfast										<0.0001
Yes	116,319	31,157	(26.8)	21,695	(18.6)	23,661	(20.4)	39,806	(34.2)	
No	33,706	9471	(28.1)	6129	(18.2)	6571	(19.5)	11,535	(34.2)	
Fast food consumption										0.1597
Frequent	125,049	33,762	(27.0)	23,256	(18.6)	25,288	(20.2)	42,743	(34.2)	
Non-frequent	24,976	6866	(27.5)	4568	(18.3)	4944	(19.8)	8598	(34.4)	
Alcohol consumption										0.6818
Yes	48,914	13,295	(27.2)	9131	(18.7)	9810	(20.1)	16,678	(34.0)	
No	101,111	27,333	(27.0)	18,693	(18.5)	20,422	(20.2)	34,663	(34.3)	
Smoking										<0.0001
Yes	12,934	4119	(31.8)	2449	(18.9)	2429	(18.8)	3937	(30.5)	
No	137,091	36,509	(26.6)	25,375	(18.5)	27,803	(20.3)	47,404	(34.6)	
Perceived stress										<0.0001
High	123,150	31,929	(25.9)	22,607	(18.4)	24,958	(20.3)	43,656	(35.4)	
Low	26,875	8699	(32.4)	5217	(19.4)	5274	(19.6)	7685	(28.6)	
Body mass index										<0.0001
Underweight	34,399	9582	(27.9)	6512	(18.9)	7026	(20.4)	11,279	(32.8)	
Normal	90,074	24,175	(26.8)	16,745	(18.6)	18,104	(20.1)	31,050	(34.5)	
Overweight	25,552	6871	(26.9)	4567	(17.9)	5102	(20.0)	9012	(35.2)	
Body image distortion										<0.0001
No	110,303	30,389	(27.5)	20,298	(18.4)	22,187	(20.1)	37,429	(34.0)	
Yes	39,722	10,239	(25.8)	7526	(18.9)	8045	(20.2)	13,912	(35.1)	

**Table 2:** Participant characteristics related to weekly average daily sedentary behavior for educational purposes.

Characteristic	Total	Weekly Average Daily Sedentary Time for Educational Purposes								*p*-Value
		1–240 min/d		241–360 min/d		361–480 min/d		≥481 min/d		
Sex										<0.0001
Male	76,776	22,075	(28.7)	16,806	(21.9)	16,616	(21.6)	21,279	(27.8)	
Female	73,249	12,586	(17.2)	13,690	(18.7)	18,710	(25.5)	28,263	(38.6)	
School grade										<0.0001
Middle school	81,692	22,017	(27.0)	18,425	(22.5)	21,075	(25.8)	20,175	(24.7)	
High school	68,333	12,644	(18.5)	12,071	(17.7)	14,251	(20.8)	29,367	(43.0)	
Academic achievement										<0.0001
High	58,015	9742	(16.8)	10,005	(17.2)	14,166	(24.5)	24,102	(41.5)	
Middle	45,370	10,626	(23.4)	8913	(19.6)	10,920	(24.1)	14,911	(32.9)	
Low	46,640	14,293	(30.6)	11,578	(24.8)	10,240	(22.0)	10,529	(22.6)	
Household income										<0.0001
High	62,362	13,545	(21.7)	11,525	(18.5)	14,100	(22.6)	23,192	(37.2)	
Middle	70,857	16,646	(23.5)	14,992	(21.2)	17,244	(24.3)	21,975	(31.0)	
Low	16,806	4470	(26.6)	3979	(23.7)	3982	(23.7)	4375	(26.0)	
Living with parents										<0.0001
Yes	143,279	33,183	(23.2)	29,271	(20.4)	34,105	(23.8)	46,720	(32.6)	
No	6746	1478	(21.9)	1225	(18.2)	1221	(18.1)	2822	(41.8)	
Regular breakfast										<0.0001
Yes	116,319	26,087	(22.4)	23,319	(20.1)	27,538	(23.7)	39,375	(33.8)	
No	33,706	8574	(25.4)	7177	(21.3)	7788	(23.1)	10,167	(30.2)	
Fast food consumption										<0.0001
Frequent	125,049	29,052	(23.2)	25,587	(20.5)	29,635	(23.7)	40,775	(32.6)	
Non-frequent	24,976	5609	(22.5)	4909	(19.6)	5691	(22.8)	8767	(35.1)	
Alcohol consumption										<0.0001
Yes	48,914	11,864	(24.2)	10,694	(21.7)	11,087	(22.7)	15,269	(31.2)	
No	101,111	22,797	(22.5)	19,802	(19.6)	24,239	(24.0)	34,273	(33.9)	
Smoking										<0.0001
Yes	12,934	3756	(29.0)	3125	(24.2)	2727	(21.1)	3326	(25.7)	
No	137,091	30,905	(22.5)	27,371	(20.0)	32,599	(23.8)	46,216	(33.7)	
Perceived stress										<0.0001
High	123,150	27,277	(22.2)	24,462	(19.9)	28,993	(23.5)	42,418	(34.4)	
Low	26,875	7384	(27.5)	6034	(22.4)	6333	(23.6)	7124	(26.5)	
Body mass index										<0.0001
Underweight	34,399	7947	(23.1)	7165	(20.8)	8465	(24.6)	10,822	(31.5)	
Normal	90,074	20,317	(22.5)	17,892	(19.9)	21,145	(23.5)	30,720	(34.1)	
Overweight	25,552	6397	(25.0)	5439	(21.3)	5716	(223.4)	8000	(31.3)	
Body image distortion										<0.0001
No	110,303	26,097	(23.6)	22,576	(20.5)	25,553	(23.2)	36,077	(32.7)	
Yes	39,722	8564	(21.6)	7920	(19.9)	9773	(24.6)	13,465	(33.9)	

**Table 3:** Participant characteristics related to weekly average daily sedentary behavior for non-educational purposes.

Characteristic	Total	Weekly Average Daily Sedentary Time for Non-Educational Purposes								*p*-Value
		1–120 min/d		121–180 min/d		181–300 min/d		≥301 min/d		
Sex										<0.0001
Male	76,776	13,890	(18.1)	16,937	(22.1)	24,603	(32.0)	21,346	(27.8)	
Female	73,249	14,575	(19.9)	16,853	(23.0)	23,260	(31.8)	18,561	(25.3)	
School grade										<0.0001
Middle school	81,692	15,212	(18.6)	17,553	(21.5)	26,265	(32.2)	22,662	(27.7)	
High school	68,333	13,253	(19.4)	16,237	(23.8)	21,598	(31.6)	17,245	(25.2)	
Academic achievement										<0.0001
High	58,015	13,046	(22.5)	14,550	(25.1)	18,391	(31.7)	12,028	(20.7)	
Middle	45,370	8133	(18.0)	10,464	(23.0)	14,933	(32.9)	11,840	(26.1)	
Low	46,640	7286	(15.6)	8776	(18.8)	14,539	(31.2)	16,039	(34.4)	
Household income										<0.0001
High	62,362	12,954	(20.8)	14,999	(24.0)	19,742	(31.7)	14,667	(23.5)	
Middle	70,857	12,512	(17.7)	15,504	(21.9)	22,970	(32.4)	19,871	(28.0)	
Low	16,806	2999	(17.8)	3287	(19.6)	5151	(30.7)	5369	(31.9)	
Living with parents										<0.0001
Yes	143,279	26,787	(18.7)	32,258	(22.5)	45,899	(32.0)	38,335	(26.8)	
No	6746	1678	(24.9)	1532	(22.7)	1964	(29.1)	1572	(23.3)	
Regular breakfast										<0.0001
Yes	116,319	22,418	(19.3)	26,981	(23.2)	37,387	(32.1)	29,533	(25.4)	
No	33,706	6047	(17.9)	6809	(20.2)	10,476	(31.1)	10,374	(30.8)	
Fast food consumption										<0.0001
Frequent	125,049	22,785	(18.2)	28,060	(22.4)	40,242	(32.2)	33,962	(27.2)	
Non-frequent	24,976	5680	(22.8)	5730	(22.9)	7621	(30.5)	5945	(23.8)	
Alcohol consumption										<0.0001
Yes	48,914	8595	(17.6)	10,330	(21.1)	15,540	(31.8)	14,449	(29.5)	
No	101,111	19,870	(19.6)	23,460	(23.2)	32,323	(32.0)	25,458	(25.2)	
Smoking										<0.0001
Yes	12,934	2377	(18.4)	2588	(20.0)	3906	(30.2)	4063	(31.4)	
No	137,091	26,088	(19.0)	31,202	(22.8)	43,957	(32.1)	35,844	(26.1)	
Perceived stress										0.0006
High	123,150	23,127	(18.8)	27,827	(22.6)	39,342	(31.9)	32,854	(26.7)	
Low	26,875	5338	(19.9)	5963	(22.2)	8521	(31.7)	7053	(26.2)	
Body mass index										<0.0001
Underweight	34,399	6736	(19.6)	7634	(22.2)	10,906	(31.7)	9123	(26.5)	
Normal	90,074	17,571	(19.5)	20,809	(23.1)	28,721	(31.9)	22,973	(25.5)	
Overweight	25,552	4158	(16.3)	5347	(20.9)	8236	(32.2)	7811	(30.6)	
Body image distortion										0.0059
No	110,303	20,724	(18.8)	24,971	(22.6)	35,327	(32.0)	29,281	(26.6)	
Yes	39,722	7741	(19.5)	8819	(22.2)	12,536	(31.6)	10,626	(26.7)	

**Table 4:** Results of logistic regression between sedentary time and body image distortion stratified by sex.

Sedentary Time (min/d)		Odds Ratio (95% Confidence Interval) for Body Image Distortion		
		Any Purpose	Educational Purposes	Non-Educational Purposes
Total	Low	Reference	Reference	Reference
	High	1.03 (1.01–1.06)	1.05 (1.02–1.08)	1.00 (0.97–1.02)
Male	Low	Reference	Reference	Reference
	High	1.01 (0.97–1.06)	1.04 (0.99–1.08)	0.99 (0.95–1.03)
Female	Low	Reference	Reference	Reference
	High	1.04 (1.01–1.08)	1.06 (1.02–1.09)	1.00 (0.97–1.04)

Results were adjusted for sex, school grade, academic achievement, household income, living with parents, regular breakfast, fast food consumption, alcohol consumption, smoking, perceived stress, and BMI. Sedentary time was categorized into low (Q1–Q3) and high (Q4) based on quartile distribution.

## Data Availability

The data used for the present work are available at the following link: https://www.kdca.go.kr/yhs/yhs/main.do (accessed on 3 May 2025).
